# Variation in the social composition of the UK academic elite: The underlay of the two—or three—cultures?

**DOI:** 10.1111/1468-4446.13154

**Published:** 2024-10-22

**Authors:** Erzsébet Bukodi, John H. Goldthorpe

**Affiliations:** ^1^ Department of Social Policy and Intervention Nuffield College University of Oxford Oxford UK; ^2^ Nuffield College University of Oxford Oxford UK

**Keywords:** education, elites, social mobility, social origins

## Abstract

In this paper, we complement a previous study of the UK natural science elite, as represented by Fellows of the Royal Society, with a comparable study of the humanities and social sciences elites, as represented by Fellows of the British Academy. We seek to establish how far similarities and differences exist in the social composition of these three academic elites and in the routes that their members have followed into elite positions. We are also concerned with the consequences of the humanities and social sciences elites being brought together in the British Academy, in contrast with the situation in most other countries where elite natural and social scientists are located in the same academy. We pursue these issues in the context of C. P. Snow's discussion of the social underlay of the cultural disjunction that he saw between the natural sciences and the humanities, while also considering how the social sciences fit in. We find that there is support for Snow's position at the time of his writing. However, a notable development in more recent years is that the growing social sciences elite is moving in its social composition away from the humanities elite and closer to the natural science elite. This is primarily due to changes in the social origins and education of Fellows in those sections of the British Academy that are on the borderline between the social and the natural sciences. A widening difference thus arises with Fellows in the humanities sections most representative of Snow's ‘traditional culture’.

## 1 INTRODUCTION

In previous papers (Bukodi et al., 2022, 2023), we have reported results from a prosopographical study—a collective biography—of the UK scientific elite as represented by Fellows of the Royal Society born since 1900.^1^ Specifically, we have provided detailed analyses of Fellows' social origins, schooling and university careers and of the routes they followed into the elite. In these papers, we are concerned with the elite in the *natural sciences.* We have subsequently undertaken a closely similar study of Fellows of the British Academy which, according to its Charter, is charged with electing to Fellowships those who have ‘attained distinction’ in the *humanities* and in the *social sciences.*

As the foregoing indicates, we have followed the practice that we have previously recommended (Bukodi & Goldthorpe, 2021) of defining membership of elites by reference to some form of institutional or associational affiliation. In this way, judgments on which individuals should be included in elites are not made subjectively by researchers, as, say, according to ‘reputation’, nor is there reliance on the, to some extent, unknown criteria used in compilations such as *Who's Who*. Furthermore, lists of names become available on the basis of which precise assessments can be made of the extent to which prosoprographical coverage of the elite in question has been achieved.^2^

Following on our work on Fellows of the British Academy, we are then in a position to undertake a *three‐way* comparison of the social composition of the elites that have been institutionally formed within UK academia as a whole through the second half of the twentieth century and into the twenty‐first.^3^ In so doing, we have two main aims.

First, we seek to establish how far similarities and differences exist in the social composition of the natural sciences, humanities and social science elites and in routes of entry into them. This focus on social composition has to be seen as distinct from—even if evidently related to—one on questions of social inequalities or ‘skewness’ in the recruitment to these elites, which would call for different research procedures from those followed here (see further Bukodi & Goldthorpe, 2021). In the case of Fellows of the Royal Society, we found an increasing homogeneity in their social origins, with a growing proportion—rising to over two‐fifths of all—coming from higher professional families, while across successive birth cohorts almost half of those educated in the UK were privately schooled. Yet, although a ‘royal road’ into the elite could be identified, via private schooling and a university career at Cambridge, still the most frequently followed route was that via state schooling and undergraduate and postgraduate attendance at UK universities outside of the ‘golden triangle’ of Cambridge, Oxford and London. Is, then, the situation that we have described in the case of the natural sciences elite essentially matched by that which exists with the humanities and social sciences elites or are there significant differences?

The results we report in this regard will form part of a wider study of UK elites in which we are engaged, covering also the political, business and civil service elites (for results on the political elite, see Bukodi et al., 2024). An ultimate concern will be with the implications of the social composition of these elites for their capacity for effective interaction.

Second, we wish to place our findings in the context of the division of responsibility for the constitution of the UK academic elite that exists between the Royal Society and the British Academy, and, in particular, as regards the allocation of the social sciences to the latter body. Cross‐nationally, a far more common arrangement is for the natural and the social sciences to be covered by a single academy, while one or more other academies cover the humanities, or for there to be one all‐inclusive academy.^4^ In the distinctive case of the UK, it is therefore of particular interest to ask the following questions. Insofar as there are differences in the social composition of the natural scientific and the humanities elites, where does the social sciences elite fit in? Is this elite more similar in its social composition to the humanities elite along with which in the UK it is institutionally included or to the natural sciences elite from which it is institutionally excluded?

In seeking to give answers to these two sets of questions, we will need—in the light of our previous study of the Fellows of the Royal Society—often to move down from the level of the elites considered as uniform entities to take account of differences in social composition that exist *within* them. That is, among their different sections as defined by particular fields of study.

## 2 RESEARCH CONTEXT: THE ‘TWO CULTURES’ CONTROVERSY

Research into academic elites of the kind in which we engage would seem to have been so far little pursued. Enquiries have been undertaken—with widely differing objectives—that have provided information, though often limited in detail or by period, on the social origins and education of academics, but of professoriates or of faculty at large rather than of some more narrowly defined elite (see, e.g., for the UK, Halsey & Trow, 1971; for France (i.e., Paris) Bourdieu, 1988; for the US, Lipset, 1982; Morgan et al., 2022). From our standpoint (Bukodi & Goldthorpe, 2021) such studies would have to be regarded as relating to the ‘pool’ from which academic elites are recruited, and as thus being of importance primarily to questions of social skewness in such recruitment: that is, how far does skewness arise in movement into the elite from the pool or from movement into the pool from the population at large? But, by the same token, it would obviously be mistaken to suppose any similarities in social composition—our focus here—as between pool and elite. We are not in fact aware of any other systematic research so far undertaken that is centred on the social composition of different elements *within* what could be regarded as a national academic elite and that extends over a lengthy period of time and across the full disciplinary range.^5^ There are thus no existing theoretical expectations towards which our research could be oriented.

This being so, and also in view of our concern with the institutional distinctiveness of the British case, we believe that our research questions and our empirical findings can be most relevantly and revealingly considered in the context of the controversy over ‘the two cultures’, initiated by C. P. Snow in 1962. This remains a controversy of some resonance, in Britain and further afield, and both academically (James, 2016; Ortolano, 2009, 2016; Whelan, 2009) and in relation to educational and research policy (Imafidon & Black, 2023; Mandler, 2015). Snow did not base his views on any systematic research but on his knowledge of scientists as a physicist‐turned‐civil servant, engaged for several years in scientific recruitment,^6^ and as a novelist and critic moving in London literary circles.

Snow's initial argument, put forward in a prestigious lecture (1962), centred on what he saw as the deep division existing between the culture of natural scientists and that of ‘literary intellectuals’ and of ‘arts people’ more generally. He emphasised differences in their ‘mental development’ and in their worldviews, values and attitudes. The scientists were optimistic, positive and with ‘the future in their bones’ while the *litterateurs* were ‘natural Luddites’, backward‐looking and, in some cases, dangerous reactionaries (1962: 10, 21‐2). Further, though, and of main interest here, Snow went on to make it clear (1964: 64) that in speaking of culture he intended its ‘anthropological’ sense—that is, as referring to a shared social environment and way of life that formed the matrix of differences of outlook. Already in journalistic pieces written before his lecture, he had in fact claimed that leading scientists tended to be men of modest social origins who had made their way through scholarships to grammar schools and to provincial universities, rather than through private schooling and then Oxford and Cambridge (Ortolano, 2009, p. 54).^7^ And in his lecture he maintained that ‘Compared with the rest of the intellectual world, considerably more scientists in this country and probably in the US come from poor families’ (1962: 10). He also cited (1962: n. 5) an unpublished study of various British elites that found that Fellows of the Royal Society were clearly more likely than leading figures in the Foreign Office or Queen's Counsel to have attended state schools.^8^

Snow had initially little to say about social scientists. But subsequently (1964: 70–71), following criticism of this omission (Burnett, 1999), he suggested that among those concerned ‘not in terms of legend but of fact’ with ‘how human beings are living or have lived’ and who are ‘on speaking terms’ with natural scientists, one could see the possible emergence of a third culture. And the bearers of this culture, he implied, would be socially closer to natural scientists than to individuals in the literary world.^9^

What then we wish to ask is how much support for what might be described as Snow's informed speculations is to be found at an elite level: that is, if we compare what we know from our previous research about the social origins and education of scientists who are Fellows of the Royal Society with the comparable information we now have on elites in the humanities and in the social sciences, as represented by Fellows of the British Academy.

As an essential preliminary, again given our interest in the possible consequences of the British institutional context, we provide a brief account of how it came about that, at the beginning of the twentieth century, the British Academy was brought into being alongside the Royal Society, founded in 1662, and that the social sciences came to be placed under the Academy's auspices. We then describe how we collected information on the social origins, schooling and university careers of all Fellows of the Academy born since 1900, following as closely as possible the procedures we used in collecting similar data for Fellows of the Royal Society. In subsequent sections, we present our findings in relation to Snow's position and in a final section we aim to draw some conclusions, including about the degree of distinctiveness of the UK case and its possible implications.

## 3 THE BRITISH ACADEMY

At a meeting in Wiesbaden in 1899 leading figures from the chief European and US learned academies, drew up plans for an international consortium of such academies, which was to be divided into two sections, Natural Sciences and Literary Sciences. At the first meeting of what had become the International Association of Academies (IAA), held in Paris in 1900, Britain was represented in the Natural Sciences section by the Royal Society but, to the concern of the organisers, had no representation in the Literary Sciences section. The Royal Society had in fact already sent a letter to several eminent persons in the humanities in Britain pointing out that this problem would arise and calling for some solution. In the outcome, two possible ways ahead were recognised: either the Royal Society would enlarge its scope, or a new body would have to be created, complementary to the Royal Society so far as participation in the IAA was concerned. A general meeting of the Fellows of the Royal Society in 1901 came out strongly in favour of the latter option. In the sequel, prominent academics from all humanities disciplines convened in the British Museum and agreed that around 70 persons should be invited to become the founding members of a new body to be called The British Academy for the Promotion of Historical, Philosophical and Philological Studies. Once thus formed, the Academy, with the support of the Royal Society, petitioned for a Royal Charter, which was granted in 1902.^10^

As regards the social sciences, at this time rather poorly developed in the UK, these were taken to be the concern of the new Academy with, it would seem, remarkably little deliberation on the matter. In its early days, the Royal Society did not limit its Fellowships to those who would today be described as natural rather than social scientists.^11^ For example, Fellows elected in 1662, as the Society came into being, included William Petty, now thought of as a pioneer of political economy, and John Graunt, acknowledged as the founder of demography (Glass, 1963; Stone, 1997) and, it might be said (Goldthorpe, 2021), also of quantitative sociology.^12^ However, over the course of the nineteenth century, the Royal Society increasingly restricted its Fellowship to those who were working in what came to be recognised as the natural, as distinct from the social—or in the more common terminology of the day—the ‘moral’ sciences.

Within the British Academy, the social science component was initially very small, amounting to little more than a few economists who were brought together in ‘a marriage of convenience’ (Winch, 2014, p. 753) with a larger number of lawyers in a section for Jurisprudence and Economics.^13^ An ‘amicable divorce’ (Runciman, 2005, p. 2) came in 1919, but it was not until 1966 that a separate section for Social and Political Studies was created, which was itself divided only in 1983, with further divisions then coming in 1999. Until late in the twentieth century, social scientists did in fact remain clearly minoritarian within the Academy. Only during the 1990s did the difference in the numbers of humanists and social scientists annually elected to Fellowships begin to narrow, before coming close to parity by the millennium (see Appendix 1).^14^ The division between the humanities and the social sciences within the Academy is explicitly recognised. The former are now subdivided into 13 sections, and the latter into eight sections. Discussions from time to time occur between the Academy and the Royal Society over where, so far as elections are concerned, the borderline between the natural and the social sciences should be drawn, as in such fields as anthropology, psychology, demography and statistics.^15^

**FIGURE 1:**
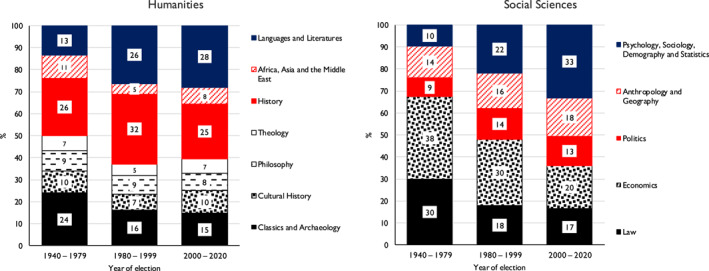
Distribution (%) of Fellows by section *within* the Humanities and Social Sciences divisions of the British Academy, by year of election.

In Figure 1 we use collapses of the Academy sections (see Appendix 3) to show how the extent of representation in the Academy of broad fields of study, within both the humanities and the social sciences, has changed over time. As is apparent, within the humanities change mainly occurs in that there is now greater representation for Languages and Literatures and less for Classics and Archaeology. Within the social sciences, change is more marked. The representation of Psychology, Sociology, Demography and Statistics increases, while there are falls in the cases of Economics and Law.

## 4 DATA

Our target population of Fellows of the British Academy was all those born since 1900, excluding Corresponding and Honorary Fellows and Fellows who, from the information available to us, had or have spent most of their working lives outside of the UK. This population then numbered 1855. We sought to obtain data on these Fellows in a variety of ways. For deceased Fellows, we rely mainly on the Academy's *Memoirs* and on the *Dictionary of National Biography* but also resort to obituaries and to other material mostly available on the web. For living Fellows, our information comes mainly from an online questionnaire sent to all of these Fellows in late 2022 and early 2023, with a response rate of 63%. But we also draw, especially in regard to university education, on *Who's Who* ^16^ and Debrett's *People of Today* and again on web material, such as interviews and news items. Full details of primary sources are given in Appendix 4.

The extent to which we were successful in acquiring information of interest to us is shown, in relation to birth cohort, in Table 1. Overall, we achieved a 78% coverage of our target population—slightly below that of the 80% achieved with Fellows of the Royal Society (Bukodi et al., 2022). Missing data is most marked in the case of parental occupation and schooling, and especially for living Fellows in more recent birth cohorts. However, analyses shown in Appendix 5, in which we investigate how far differences arise in the case of the items in question as between living and deceased Fellows within the same birth cohort indicate that the differences are small and on no evident pattern. We do therefore believe it defensible for us to take the Fellows on whom we do have information as being adequately descriptive of our target population, even if based on a somewhat incomplete set of observations. Where we resort to statistical modelling, we do not therefore apply tests of statistical significance but focus on the pattern and size of the coefficients returned.^17^

**TABLE 1:** Extent of coverage of information on social characteristics of target population of British Academy Fellows, by birth cohort.

	Birth cohort							All
	1900–09	1910–19	1920–29	1930–39	1940–49	1950–59	1960‐	
All								
Initial target population (*N*)	179	209	218	268	448	328	205	1855
% of missing information on								
parental occupation	1.1	4.8	8.7	25.0	25.9	31.7	29.8	20.4
type of secondary school	1.1	1.4	2.3	3.4	11.4	17.1	20.0	9.0
university	0.0	0.0	0.0	0.4	0.5	1.2	2.4	0.7
Cumulative	2.2	5.7	10.1	26.5	27.0	34.2	32.7	22.1
Achieved target population								
*N*	175	197	196	197	327	216	138	1446
%	97.8	94.3	89.9	73.5	73.0	65.9	67.3	78.0
Humanities								
Initial target population (*N*)	138	166	166	183	255	178	97	1183
% of missing information on								
parental occupation	1.5	5.4	9.0	27.9	27.1	30.3	24.7	18.9
type of secondary school	0.0	1.2	2.4	3.8	11.0	15.2	15.5	7.0
university	0.0	0.0	0.0	0.6	0.8	0.6	3.1	0.6
Cumulative	1.5	6.0	10.8	29.5	28.2	33.2	27.8	20.5
Achieved target population								
*N*	136	156	148	129	183	119	70	941
%	98.6	94.0	89.2	70.5	71.8	66.9	72.2	79.5
Social sciences								
Initial target population (*N*)	41	43	52	85	193	150	108	672
% of missing information on								
parental occupation	0.0	2.3	7.7	18.8	24.4	33.3	34.3	23.1
type of secondary school	4.9	2.3	1.9	2.4	11.9	19.3	24.1	12.5
university	0.0	0.0	0.0	0.0	0.0	2.0	1.9	0.8
Cumulative	4.9	4.7	7.7	20.0	25.4	35.3	37.0	24.9
Achieved target population								
*N*	39	41	48	68	144	97	68	505
%	95.1	95.4	92.3	80.0	74.6	64.7	63.0	75.2

In the study of Fellows of the Royal Society, the number of women in our target population was only 155, or around 7% of the total, and we obtained adequate information on 125 of these women—the same 80% level of coverage as with men. This number was too small to allow for consideration of gender differences other than of a very simple kind. In the case of Fellows of the British Academy, the number of women in our target population is 355, or 19% of the total, although, as earlier noted, with a heavy concentration in more recent birth cohorts. We have full information on 257 of these women, representing a 72% coverage as against 79% for men. Details can be found in Appendix 6. We have included gender in all statistical modelling that we have undertaken, but for the most part, gender differences prove in fact to be relatively small.

## 5 RESULTS

### 5.1 Social origins

We treat social origins in terms of parental social class, and we categorise class positions according to a version of the National Statistics Socio‐Economic Classification (Office of National Statistics, 2005) based on occupation and employment status, as shown in Table 2.^18^

**TABLE 2:** Modified version of National Statistics Socio‐Economic Classification (NS‐SEC).

	NS‐SEC		Modified version and labelling used
1	Large employers^a^ higher managers and higher professionals	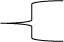	Large employers and higher managerial^b^
			Higher professional^b^
2	Lower managers, higher supervisors, lower professionals and higher technicians	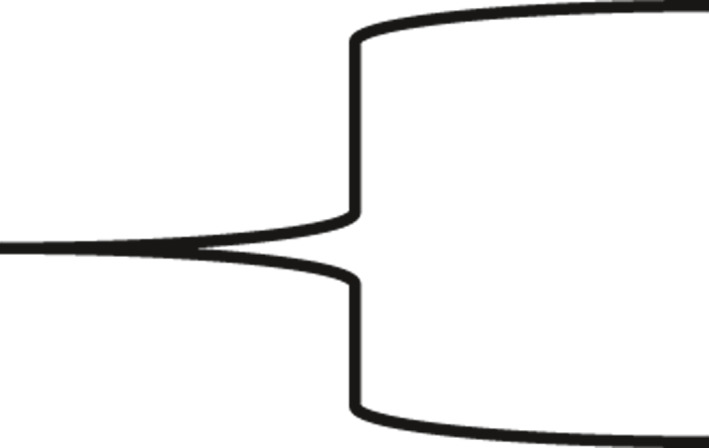	Lower managerial^b^
			Lower professional^b^
3 5	Intermediate, clerical etc. employees Lower supervisors and technicians	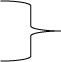	Intermediate
4	Small employers and own account workers		Self‐employed
6 7	Semi‐routine workers Routine workers	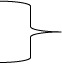	Working class

^a^ With more than 25 employees.

^b^ We follow here the NS‐SEC distinction between Class 1.1 and 1.2 and make a similar distinction within Class 2.

In Table 3, we set out relevant distributions of class origins. In the first three columns, we compare distributions for individuals in the three elites we consider, taken in their entirety. From the first two columns, it can be seen, that individuals in the natural sciences elite are less likely to be of Class 1 origins than are those in the humanities elite—45% as against 57%—and mainly because of the far smaller proportion coming from the families of large employers and higher managers. Offsetting this, the natural science elite is more likely to be recruited from Class 4 and from Class 6 and 7 families—those of small employers, self‐employed workers or, mainly manual, wage‐workers. Over a fifth of this elite are of such origins as against little more than a 10th of the humanities elite. As regards the social sciences elite, what emerges is that in the distribution of the class origins of its members it falls in‐between what we find for the two other elites.

**TABLE 3:** Distribution (%) of members of three elites by parental class.

Class of origin	Natural sciences	Humanities	Social sciences	Sections within humanities^a^							Sections within social sciences^b^				
				[1]	[2]	[3]	[4]	[5]	[6]	[7]	[1]	[2]	[3]	[4]	[5]
Class 1	45	57	49	67	65	71	50	50	51	52	54	59	51	39	41
Large employers and managerial	13	23	20	28	26	24	11	22	26	19	25	25	28	10	15
Professional	32	34	28	38	39	47	39	28	25	33	29	33	23	29	26
Class 2	22	23	23	20	25	20	29	25	25	23	28	16	27	26	22
Managerial	8	5	6	6	6	4	6	6	1	6	10	6	6	6	5
Professional	14	18	17	14	19	16	23	18	24	18	18	10	21	20	17
Classes 3 and 5	10	9	12	7	6	4	13	12	13	9	7	11	8	15	16
Class 4	12	6	11	3	3	4	3	9	7	7	8	13	10	13	12
Classes 6 and 7	11	5	5	3	1	2	5	5	3	9	3	2	4	7	10
Total	100	100	100	100	100	100	100	100	100	100	100	100	100	100	100
*N*	1726	955	514	172	89	85	62	265	68	214	100	126	71	89	128

^a^ [1] Classics and Archaeology; [2] Cultural History; [3] Philosophy; [4] Theology; [5] History; [6] Africa, Asia and the Middle East; [7] Languages and Literatures.

^b^ [1] Law; [2] Economics; [3] Politics; [4] Anthropology and Geography; [5] Psychology, Sociology, Demography and Statistics.

A fuller understanding of this situation can, however, be gained from the further columns of Table 3, in which we show the class origins distributions of those in different sections of the humanities and social sciences elites.^19^ Across the sections, in both elites, the most common pattern is for around 50%–55% of their members to come from Class 1 origins and for 10%–15% to come from Class 4 and Class 6 and 7 origins. But in both cases, there are some notable deviations. Within the humanities elite, in three sections, Classics and Archaeology, Cultural History and Philosophy, 65% or more of their members are of Class 1 origins, while those from Class 4 and Class 6 and 7 origins range from only 4%–6%. In the social sciences elite, in contrast, there are two sections, Anthropology and Geography and Psychology, Sociology, Demography and Statistics, where members of Class 1 origins amount to only around 40% of all, with over 20% coming from Class 4 and Class 6 and 7 origins.

What is then of interest is that it is with these two latter sections of the Academy, where the borderline with the natural sciences is least clear, that the class origins of Fellows come into the closest correspondence with those of Fellows of the Royal Society. If, from Table 3, we calculate the dissimilarity indices that result from comparing the relevant distributions of class origins, we find that in the case of the two sections in question these amount to only 12.0 and 12.6, respectively. That is to say, only 12–13% of their distributions would have to be ‘transferred’ to that of the Fellows of the Royal Society to make them identical. The corresponding indices in the case of Fellows in the Classics and Archaeology, Cultural History and Philosophy sections of the Academy are 25.0, 22.0 and 28.0, respectively.

**FIGURE 2:**
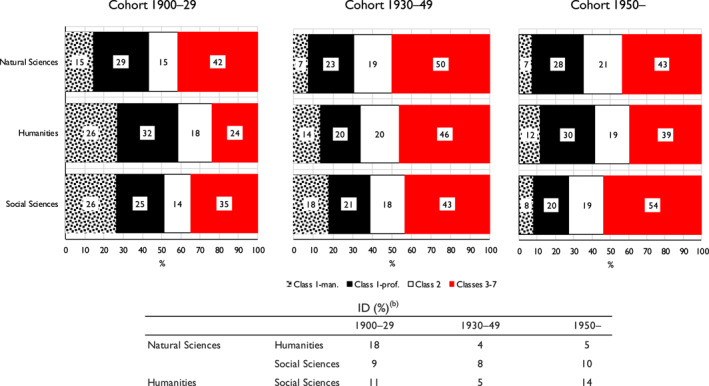
Expected probability (%) of members of three elites having come from different classes of origin, and indices of dissimilarity [ID], by birth cohort.^a^ ^a^Based on Appendix 7, Model 2. ^b^Based on a three‐fold classification: Class 1; Class 2; Classes 3–7.

Finally, it is important to consider changes over time—that is, across the birth cohorts that we distinguish. To do this, we take an ‘inflow’ perspective and run a multinomial logit model in which the dependent variable is the probability of elite individuals having come from different classes of origin, with the explanatory variables being membership of one or other of our three elites and birth cohort, together with interactions between them. Gender is also included in the model. To maintain adequate numbers, we now collapse Classes 3–7 and the six cohorts of Table 1 to three. Full details of the model and of all results from it are given in Appendix 7.1 (Model 2). The main results are presented here in Figure 2.

As is apparent, for the earliest cohort—those born 1900‐29—the pattern revealed is on much the same lines as was found when the three elites were considered across all cohorts. It is with the humanities elite that the probability of individuals coming from Class 1 origins is highest and the probability of their coming from Classes 3–7 origins lowest. The reverse is the case with the natural sciences elite, and the social sciences elite falls in‐between.

For the second cohort, those born 1930–49, the differences across elites in class of origin are reduced—the dissimilarity indices for the probabilities that are shown all fall. This results from a general increase—but one most marked within the humanities—in the probability of elite members coming from relatively disadvantaged, Class 3–7, origins. This trend is consistent with the rising chances of upward mobility for those born around this time within the population at large (Bukodi & Goldthorpe, 2019: ch. 2).

However, with the third cohort, those born from 1950 on, differences between the elites widen again, and it is now the social sciences elite that becomes distinctive. While in both the natural sciences and humanities elites the proportion of those coming from Class 3–7 origins *decreases*, as compared with the second cohort, in the social sciences elite the increase in this proportion between the first and second cohorts continues into the third. With this cohort, a clearly lower probability is then evident of members of the social sciences elite coming from Class 1 origins, and especially from Class 1 professional origins, as compared with members of the other two elites, and a higher probability of their coming from less advantaged origins.^20^

The results of similar modelling relating to different sections of the social science elites, though with some further collapsing, show (Appendix 8.3–8.4) that it is in fact in the Anthropology and Geography and in the Psychology, Sociology, Demography and Statistics sections of the Academy that the increase in those coming from Classes 3–7 origins is strongest. For members of these sections born after 1950, there is a 57% probability of their being of such origins as against only a 23% probability of their being of Class I origins.

It would then appear that differences in class origins as between members of the natural sciences and humanities elites were, at the time of Snow's writing, largely on the lines that he envisaged. But what is of further interest is that while subsequently these differences have narrowed somewhat, the social sciences elite has become overall more differentiated in its class composition from the two other elites, although with Fellows in certain sections bordering on the natural sciences coming closer in this regard to the natural sciences elite.

### 5.2 Secondary schooling

Given that a strong association is known to exist between class origins and type of schooling, one might expect that differences emerging among our three elites in regard to their schooling will be on much the same lines as those found with their class origins. But, while this is broadly the case, there are some variations of note.

In Table 4, we show distributions for the three elites by type of secondary schooling, distinguishing between categories of private and state schools and schooling abroad. From the first two columns of the table, it can indeed be seen that those in the humanities elite, as well as being more likely to come from Class 1 origins than those in the natural sciences elite, are also more likely to have been privately rather than state schooled—50% as against 37%. And, within the private sector, they are more likely to have attended Clarendon^21^ or other boarding schools rather than day schools. Further, though, when in the third column of the table we come to the social sciences elite, we find that the members of this elite, rather than falling into an intermediate position, as with their class origins, are in their schooling very similar to members of the natural sciences elite. The dissimilarity index between the two distributions is only 10.

**TABLE 4:** Distribution (%) of members of three elites by type of secondary school.

Type of secondary school	Natural sciences	Humanities	Social sciences	Sections within humanities^a^							Sections within social sciences^b^				
				[1]	[2]	[3]	[4]	[5]	[6]	[7]	[1]	[2]	[3]	[4]	[5]
Private	37	50	34	59	58	55	54	47	44	43	40	33	39	29	30
Clarendon	7	12	7	14	17	22	10	11	10	7	5	11	12	2	4
Other, boarding	19	25	15	30	30	21	21	25	20	21	23	12	19	16	11
Other, day	11	14	12	15	10	12	23	11	14	15	12	10	8	12	14
State	51	36	49	32	24	20	41	39	35	43	47	45	37	57	54
Grammar, direct grant	3	4	5	7	1	3	1	3	3	5	6	3	3	3	9
Grammar, maintained	38	27	34	19	18	14	31	31	28	33	30	38	24	41	32
Comprehensive	3	2	6	2	3	0	1	1	1	2	10	1	8	6	9
Other	6	4	4	3	2	3	7	4	4	3	2	2	3	8	5
Non‐UK	13	14	17	9	18	24	4	13	20	14	13	22	24	14	16
Total	100	100	100	100	100	100	100	100	100	100	100	100	100	100	100
*N*	1940	1100	589	195	99	94	70	314	79	249	115	147	75	103	149

^a^ [1] Classics and Archaeology; [2] Cultural History; [3] Philosophy; [4] Theology; [5] History; [6] Africa, Asia and the Middle East; [7] Languages and Literatures.

^b^ [1] Law; [2] Economics; [3] Politics; [4] Anthropology and Geography; [5] Psychology, Sociology, Demography and Statistics.

Turning to the columns of Table 4 in which we show educational distributions by sections of the humanities and social sciences elites, one can readily see differences paralleling those in class origins. The major contrasts are again between, on the humanities side, those in Classics and Archaeology, Cultural History and Philosophy and, on the social sciences side, those in Anthropology and Geography and in Psychology, Sociology, Demography and Statistics. Of the former, 55%–60% were privately schooled as against only 30% of the latter, with offsetting differences being largely in the proportions state educated.

**FIGURE 3:**
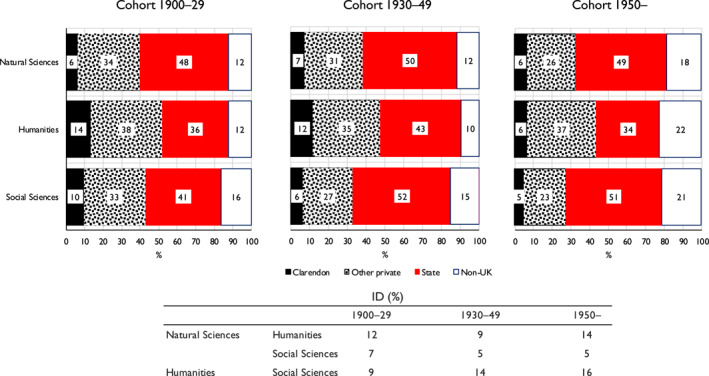
Expected probability (%) of members of three elites having attended different types of secondary school, and indices of dissimilarity [ID], by birth cohort.^a^ ^a^Based on Appendix 9, Model 2.

As regards changes over time, we again resort to multinomial logit modelling on the same lines as with class origins. The dependent variable is now the probability of elite members having attended different types of secondary school and the explanatory variables are elite membership and birth cohort (collapsed as previously) with interactions between them. However, we also include class of origin as well as gender as additional variables. Full details are given in Appendix 9. Figure 3 is based on the results there reported (Model 2).

Two features of Figure 3 are of note. First, although the probability of members of the humanities elite being privately schooled falls across the cohorts, due to a declining probability of being Clarendon educated, they remain significantly more likely than members of the two other elites to have been so schooled—43% as against around 30% in our third cohort. And there is also in this cohort a drop in the probability of their being state schooled—as there was also in the probability of their coming from relatively disadvantaged class origins. Second, the social sciences elite steadily moves away from the humanities elite as regards the probabilities of its members' schooling and comes closer to the natural sciences elite. For the most recent cohort, the social sciences elite has a dissimilarity index with the humanities elite of 16 but with the natural sciences elite of only 5.^22^ Similar modelling applied to different sections within the humanities and social science elites (Appendix 10) indicates that these two tendencies in fact show up in a fairly uniform way across their different sections.

Again, therefore, there is support for Snow in that the natural sciences elite is more likely than the humanities elite to have attended state rather than private schools. But also of interest is that the social sciences elite, rather than being in an intermediate position as with class origins, shows an increasing probability of its members being state schooled and thus becomes in this respect very similar to the natural sciences elite.

### 5.3 Universities

Differences in university attendance among the three elites we consider are again on lines that might be expected, given what we have already established regarding class origins and now also type of secondary schooling. But again, too, there are deviations.

In Table 5, we show distributions for the three elites by university or type of university attended at undergraduate level. In the light of the foregoing, it is not surprising to find that a substantial majority—65%—of members of the humanities elite were at Cambridge or Oxford, with this proportion rising to around 70% in the case of those in Classics and Archaeology and in Philosophy. In contrast, only a little over 40% of the members of the natural sciences elite and also of the social sciences elite attended Cambridge or Oxford, offset by more attending both London and universities outside of the Cambridge‐Oxford‐London golden triangle. A further relevant factor in the case of the social sciences elite is of course their relatively slow and weak development, especially in sociology and demography, at both Cambridge and Oxford until quite late in the twentieth century.

**TABLE 5:** Distribution (%) of members of three elites by undergraduate university attended.

University	Natural sciences	Humanities	Social sciences	Sections within humanities^a^							Sections within social sciences^b^				
				[1]	[2]	[3]	[4]	[5]	[6]	[7]	[1]	[2]	[3]	[4]	[5]
Oxbridge	41	65	42	69	55	72	67	73	53	56	52	42	40	44	37
Cambridge	28	29	22	31	31	21	32	29	32	28	23	26	13	29	19
Oxford	13	36	20	38	24	51	35	43	21	28	29	16	27	15	18
London	15	6	15	7	9	4	6	3	9	8	14	12	13	18	17
Other Russell group^c^	23	10	15	9	8	4	15	8	14	14	13	17	14	15	16
Other UK	8	5	9	5	8	2	6	5	1	7	6	6	12	10	13
Non‐UK	13	14	18	10	19	18	7	12	23	15	15	23	21	14	16
Total	100	100	100	100	100	100	100	100	100	100	100	100	100	100	100
*N*	2110	1166	666	210	107	99	72	320	87	271	127	168	85	108	178

^a^ [1] Classics and Archaeology; [2] Cultural History; [3] Philosophy; [4] Theology; [5] History; [6] Africa, Asia and the Middle East; [7] Languages and Literatures.

^b^ [1] Law; [2] Economics; [3] Politics; [4] Anthropology and Geography; [5] Psychology, Sociology, Demography and Statistics.

^c^ The Russell Group universities are a self‐selected group of ‘leading research universities’ in the UK, established in 1994, and now comprising 24 institutions. Cambridge and Oxford are included along with some but not all University of London colleges, many of which are now in effect independent universities in themselves.

**FIGURE 4:**
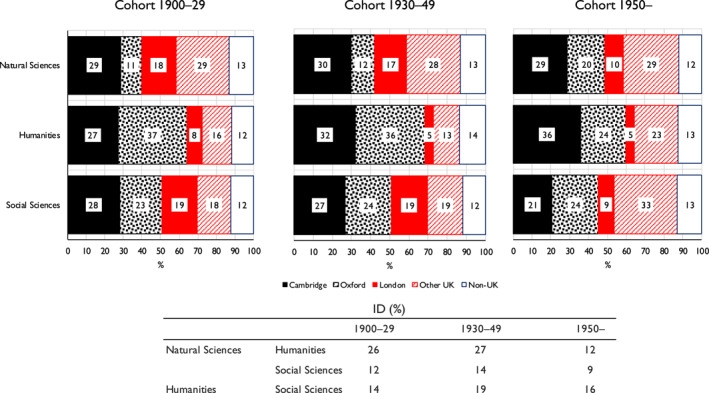
Expected probability (%) of members of three elites having attended different universities as undergraduates, and indices of dissimilarity [ID], by birth cohort.^a^ ^a^Based on Appendix 11, Model 2.

In treating changes over time, we follow the same modelling approach as previously. The probability of having attended different universities or types of university is the dependent variable, with elite membership and birth cohort being the explanatory variables and with now both type of schooling and class origins being included along with gender. Full results are given in Appendix 11 (Model 2) and Figure 4 shows the main findings.

Three points of interest emerge. First, while for the natural sciences elite, Cambridge is the pre‐eminent university (see further Bukodi et al., 2023), over the birth cohorts Oxford does to some extent catch up. But, second, within the humanities elite, there is in the most recent cohort a marked decline in the probability of its members having gone to Oxford, offset by an increase in the probability of their having gone to Cambridge and to universities outside of the golden triangle. Largely as a result of these two tendencies, in the most recent cohort the dissimilarity index for the probability distributions of the natural sciences and humanities elites falls to 12—less than half of what it was previously—even though members of the humanities elite still have the clearly higher probability of having been at Cambridge *or* Oxford. Third, in the case of the social sciences elite, there is little change in the probability of its members having attended Oxford, but in the most recent cohort there are declines in their probabilities of having been at Cambridge, and also at London, and a marked increase in the probability of their having attended universities outside of the golden triangle. While then for this cohort the dissimilarity index for the probability distributions of the social sciences and humanities elites remains high at 16, that for the social sciences and the natural sciences elites falls to 9.^23^

The results of similar modelling that includes sections mainly show that in the case of the social sciences elite (Appendix 12.3, 12.4) there is a declining probability of attendance at both Cambridge and London across the sections, while, especially in the most recent birth cohort, the probability of having been at other universities increases.

Moving on now to Table 6, relating to university education at postgraduate level, what is first of all notable is that almost a quarter of those in the humanities elite—and rising to over a third with those in Classics and Archaeology—did not undertake postgraduate work or, that is, not in any formal sense. And the same is the case with 14% in the social sciences elite. What is chiefly reflected here is the possibility offered at Cambridge and Oxford for undergraduates in the humanities and social sciences who obtained outstanding honours degrees to make their start in what might turn out to be distinguished academic careers through obtaining junior College fellowships rather than by taking a higher degree. In view of this, what perhaps most significantly emerges from the table is that less than a third of the humanities elite were postgraduates at universities *other than* Cambridge and Oxford, as compared with somewhat over a half of members of both the natural and the social sciences elites.^24^ Also of note is that within the social sciences elite, it is members of the Academy sections of Anthropology and Geography and of Psychology, Sociology, Demography and Statistics who are the *least* likely to be without a higher degree—in only 9% of cases. Again, it is these sections that come closest to what is found within the natural sciences elite in what might be regarded as the professionalisation of their disciplines, although their relatively late representation in the social sciences elite is, as will be seen, also a factor.

**TABLE 6:** Distribution (%) of members of three elites by postgraduate university attended.

University	Natural sciences	Humanities	Social sciences	Sections within humanities^a^							Sections within social sciences^b^				
				[1]	[2]	[3]	[4]	[5]	[6]	[7]	[1]	[2]	[3]	[4]	[5]
Oxbridge	38	45	33	47	17	47	54	53	38	44	44	30	34	33	26
Cambridge	26	23	14	25	12	20	26	23	21	25	15	15	8	20	13
Oxford	13	22	18	21	5	27	28	30	17	20	29	15	26	13	13
London	19	11	18	7	31	7	4	9	15	11	10	16	14	28	21
Other Russell group^c^	20	7	12	6	7	3	11	5	8	11	10	9	11	11	17
Other UK	6	3	9	1	4	1	4	2	1	5	2	4	19	10	13
Non‐UK	11	10	14	6	19	15	4	7	15	13	15	21	9	8	13
None	6	24	14	34	23	26	22	23	23	16	18	20	13	9	9
Total	100	100	100	100	100	100	100	100	100	100	100	100	100	100	100
*N*	2105	1167	664	208	107	99	72	324	87	270	125	167	85	109	178

^a^ [1] Classics and Archaeology; [2] Cultural History; [3] Philosophy; [4] Theology; [5] History; [6] Africa, Asia and the Middle East; [7] Languages and Literatures.

^b^ [1] Law; [2] Economics; [3] Politics; [4] Anthropology and Geography; [5] Psychology, Sociology, Demography and Statistics.

^c^ The Russell Group universities are a self‐selected group of ‘leading research universities’ in the UK, established in 1994, and now comprising 24 institutions. Cambridge and Oxford are included along with some but not all University of London colleges, many of which are now in effect independent universities in themselves.

**FIGURE 5:**
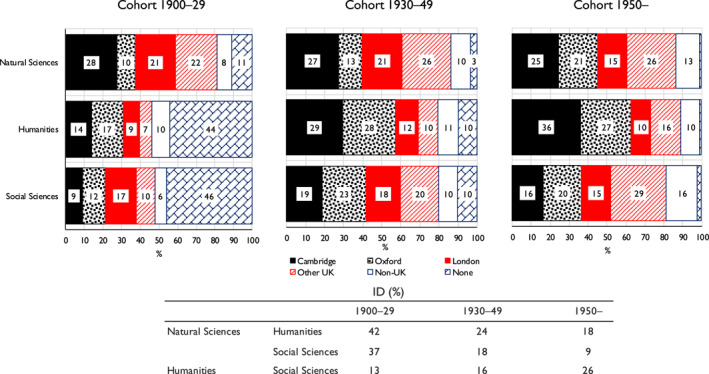
Expected probability (%) of members of three elites having attended different universities as postgraduates, and indices of dissimilarity [ID], by birth cohort.^a^ ^a^Based on Appendix 13, Model 2.

When we turn to changes over time, applying in Appendix 13 a model analogous to that of Appendix 11 but now relating to the postgraduate level, a more qualified view can be gained. From the results shown in Figure 5, it can be seen that in both the humanities and social sciences elites alike the high probability of members having no formal postgraduate education is very much a feature of the earliest cohort, those born 1900‐29. By the most recent cohort, this probability becomes negligible. Aside from this, changes are on much the same lines as those found at the undergraduate level. While Oxford is closing the gap on Cambridge within the natural sciences elite, the reverse movement occurs within the humanities elite—Cambridge moves ahead of Oxford in the most recent cohort. Thus, in this cohort there is still, as at the undergraduate level, more than a 60% probability of the humanities elite having done their postgraduate studies at Cambridge or Oxford, while the corresponding figure is significantly lower in the other two elites, and in the social sciences elite in particular. The dissimilarity indices for the probability distributions of the natural sciences elite as against both the humanities and social sciences elites fall—in the latter case to again as low as 9—but the dissimilarity index for the distributions of the humanities elite and the social sciences elite widens yet again to as much as 26.^25^

When we also consider change across sections, no major and systematic differences show up in the case of humanities elite (see Appendix 14.1, 14.2). But within the social sciences elite, it is change in the sections of Anthropology and Geography and of Psychology, Sociology, Demography and Statistics that is, once more, most marked (see Appendix 14.3, 14.4). There is a substantial increase in the probability of members of these sections having been postgraduates at universities outside of the golden triangle and mainly at the expense of London University.

In sum, in the case of university attendance, there is evidence of the division that Snow saw between the natural sciences and the humanities elites weakening somewhat, although the former remain clearly less likely than the latter to be ‘Oxbridge’ products. But a more marked change is the widening difference in the pattern of university attendance as between the humanities and the social sciences elites, despite their co‐existence within the British Academy. Over the birth cohorts we consider, university composition of the social sciences elite comes very close that of the natural sciences elite, and Snow might be given credit for some prescience insofar as this tendency is driven by those Fellows of the Academy who could be regarded as being ‘on speaking terms’ with natural scientists.

### 5.4 Routes into the three elites

We have analysed comparatively the class origins, schooling and universities of the members of our three academic elites. We now aim to bring out how the associations we have shown find expression in the actual routes that individuals have followed from their class origins via their schools and universities (excluding now those who attended non‐UK schools or universities) to their elite positions. To begin with, Table 7 sets out the educational routes of elite members, distinguishing between private and state schooling and undergraduate and postgraduate universities.

**TABLE 7:** Secondary school, undergraduate university, postgraduate university careers in three elites (%)^a^.

Secondary school	Undergraduate	Postgraduate	Natural sciences	Humanities	Social sciences
Private	Cambridge	Cambridge^b^	**14**	**17**	**9**
		Oxford	1	2	1
		London	3	1	2
		Other UK	1	1	1
	Oxford	Cambridge	1	1	0
		Oxford^b^	**6**	**26**	**11**
		London	1	2	2
		Other UK	1	0	2
	London	Cambridge	0	0	0
		Oxford	0	0	0
		London	**4**	2	2
		Other UK	1	0	1
		None	1	1	2
	Other UK	Cambridge	2	1	1
		Oxford	1	1	1
		London	1	1	1
		Other UK	**4**	2	**4**
		None	0	0	1
State	Cambridge	Cambridge^b^	**9**	**12**	**10**
		Oxford	1	1	1
		London	1	1	2
		Other UK	1	1	1
	Oxford	Cambridge	1	0	0
		Oxford^b^	**5**	**11**	**9**
		London	1	1	1
		Other UK	1	0	1
	London	Cambridge	1	0	1
		Oxford	0	0	1
		London	**8**	2	6
		Other UK	1	0	2
		None	1	0	1
	Other UK	Cambridge	3	2	1
		Oxford	1	2	2
		London	2	2	3
		Other UK	**20**	5	**13**
		None	1	1	2
Total			100	100	100
*N*			1618	880	439

^a^ Fellows who attended non‐UK schools or universities are excluded.

^b^ Fellows who did not pursue formal postgraduate studies are also included.

It should be noted, that in Table 7 we treat elite members who had no formal postgraduate qualifications but were graduates of Cambridge or Oxford as having continued with academic work at their undergraduate university. The table does in fact give a clear indication that there is in general a strong tendency for individuals to stay on as postgraduates at the same university at which they were undergraduates.^26^

Adding the figures shown in bold in Table 7 will indicate the dominant routes followed into the three elites. In the case of the natural sciences elite, the Cambridge undergraduate‐postgraduate sequence accounts for 23% of all members, the Oxford for 11%, the London for 12%, and the Other UK for 24%. With the first two of these sequences, there is a private school bias but with the latter two, a state school bias. In other words, there are four routes, one or other of which has been followed by two‐thirds of all those in this elite. In some contrast, with the humanities elite, two‐thirds of all members have followed just two routes: the Cambridge sequence, accounting for 29%, and the Oxford for 37%, with in both cases a clear private school bias. In the case of the social sciences elite a clearly greater diversity in educational routes is apparent. The Cambridge sequence accounts for 19% of all members and the Oxford sequence for 20% with little school bias either way, while, again as with the natural sciences elite, the Other UK sequence is of importance in accounting for 17%, with a clear state school bias. But, in sum, these three routes together account for only somewhat over half of the members of the social sciences elite. The remainder are quite thinly scattered over all the other possible routes shown in Table 7 with, somewhat surprisingly, the London undergraduate‐postgraduate sequence accounting for no more than 8%.

In moving on, we focus on those educational routes that, with somewhat more simplification, are the most often followed across our three elites. We maintain the private/state schooling distinction but at the university level, while keeping the Cambridge and Oxford sequences, we combine the London sequence with the Other UK sequence, thus giving what can be labelled as a non‐Oxbridge sequence. The six routes in question do in fact account for 77% of all elite members. However, for completeness, we include, also with the private/state schooling distinction, a Rest category, which comprises the remaining 23% of individuals who followed more diverse routes. What we are then concerned with is how the probabilities of members of our three elites having followed these different routes are related, first, to their class origins and, second, to birth cohort.

**FIGURE 6:**
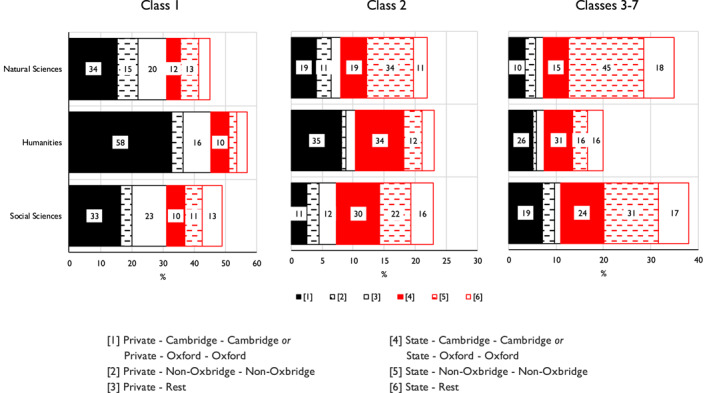
Expected probability (%) of members of three elites having followed six secondary school and undergraduate and postgraduate university careers, by class of origin.^a–c^ ^a^Fellows who attended non‐UK schools or universities are excluded. ^b^Based on Appendix 15. ^c^Length of bars drawn proportionately to number of elite members coming from each class of origin. Within bars, only proportions higher than 10% are marked.

To pursue these questions, we run the multinomial logistic model reported on in Appendix 15. As regards class origins, the main results are shown in Figure 6, in which the lengths of bars are drawn proportionately to the number of elite members coming from each class of origin. Further, to ease interpretation, we here combine in the figure the Cambridge and Oxford sequences.

As is evident, the humanities elite stands apart from the natural and social sciences elites. The higher probability of its members coming from Class 1 origins, as earlier observed, can now be seen to be linked with their higher probability of having followed routes via private schooling and undergraduate and postgraduate study at either Cambridge or Oxford. Individuals of Class 1 origins in the humanities elite have taken such a route with a 58% probability as compared with a 34% and a 33% probability for their counterparts in the natural sciences and social sciences elites. Conversely, the higher probability of members of the two latter elites coming from Classes 3–7 origins is linked to their having followed state schooling and non‐Oxbridge routes with 45% and 31% probabilities, respectively, as compared with only a 16% probability for those of Classes 3–7 origins in the humanities elite.^27^ ^,^ ^28^

**FIGURE 7:**
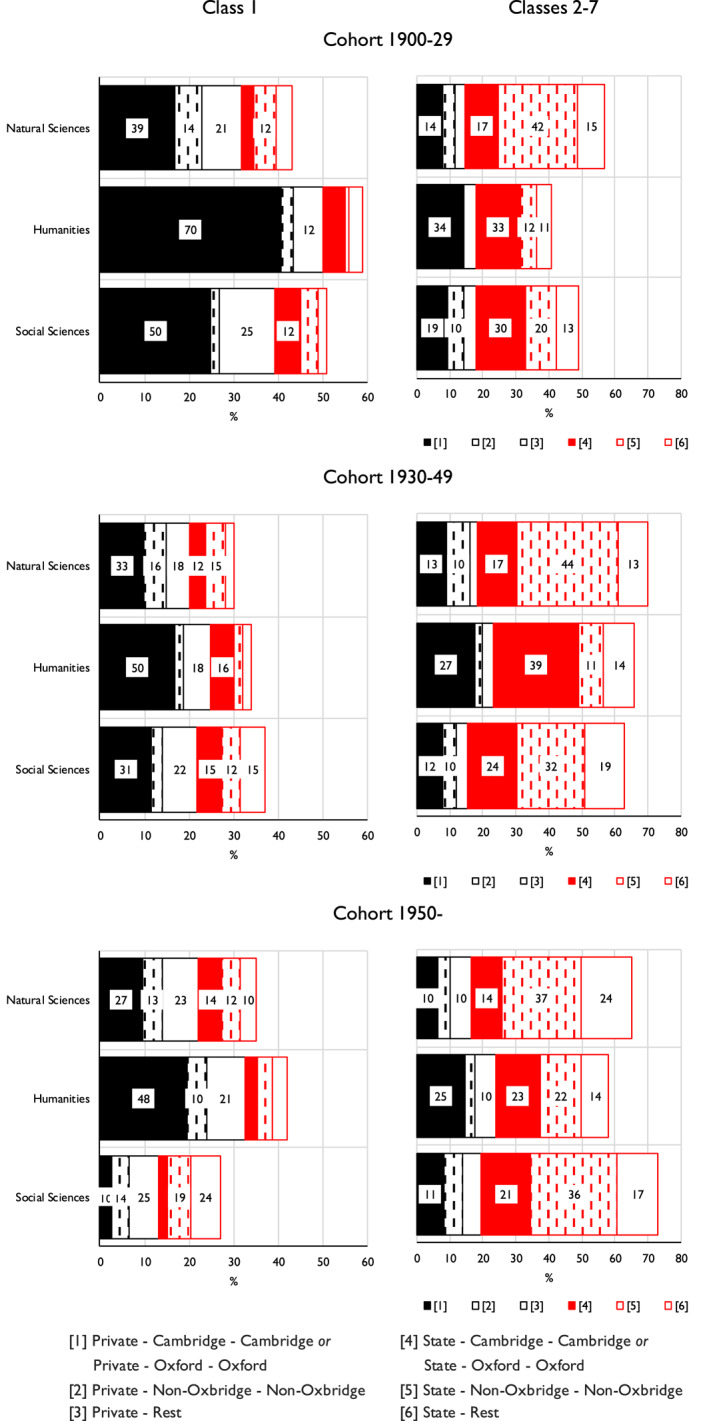
Expected probability (%) of members of three elites having followed six secondary school and undergraduate and postgraduate university careers, by class of origin and birth cohort.^a–c^ ^a^Fellows who attended non‐UK schools or universities are excluded. ^b^Based on Appendix 16. ^c^Length of bars drawn proportionately to number of elite members coming from each class of origin. Within bars, only proportions higher than 10% are marked.

How far then is this situation stable over time? In Figure 7, based on the further model of Appendix 16, we bring three birth cohorts into the analysis, while at the same time setting Class 1 origins against the rest, in order to preserve adequate numbers. As is apparent, the probability of elite members of Class 1 origins having moved on from private schooling to university careers at Cambridge or at Oxford generally declines across the cohorts. However, this decline is much more marked with the social sciences elite than with the humanities or the natural sciences elites. As a result, among those born from 1950 onwards, members of the humanities elite of Class 1 origins have still a 48% probability of having followed the route in question, as compared with a 27% probability for members of the natural sciences elite and as little as a 10% probability for members of the social sciences elite. And with those of other class origins in this same cohort, state schooling and non‐Oxbridge routes were followed with a 37% probability by members of the natural sciences elite and a 36% probability by members of the social sciences elite as compared with only a 22% probability by members of the humanities elite.

Finally, similar modelling, reported on in Appendix 17, shows that in the case of the humanities elite the prevalence of the route from private schooling to Cambridge or Oxford is quite general, though somewhat weaker in Languages and Literatures than in other sections. However, there is a sharper division within social sciences elite. For those of Class 1 origins, there is a 36% probability of the route in question being followed by members of the Academy sections of Law, Economics and Politics, as against only a 20% probability for members of the sections of Anthropology and Geography and of Psychology, Sociology, Demography and Statistics. Conversely, for those not of Class 1 origins, the probability of being state schooled and moving on to universities other than Cambridge or Oxford is 38% for members of the former sections as against 56% for members of the latter.

What we find here is then confirmation of an emergent social sciences elite, of the kind envisaged by Snow, which is formed through individuals following more diverse routes from class origins via schooling and university than is the case with the natural sciences elite and, especially, with the humanities elite. The latter is distinctive in the persisting prevalence in its formation of the route from advantaged class origins, via private schooling and Oxbridge.

## 6 CONCLUSIONS

The first aim we set out at the start of this paper was that of establishing how far similarities or differences exist in the social composition of the three academic elites that we distinguish and in the routes that their members have followed into their elite positions. We can now give a general answer in straightforward terms. Differences are more notable than similarities and fall into a well‐defined pattern.

In the comparison between the social composition of the humanities and the natural sciences elites, Snow's views relating to the 1960s turn out to be supported for that time. Fellows in the humanities division of the British Academy were more likely than Fellows of the Royal Society to be of Class 1 rather than of less advantaged class origins, to have been privately rather than state schooled, and to have attended Cambridge or Oxford rather than other universities. Such differences decrease somewhat across the birth cohorts we distinguish but still remain. However, a more complex picture emerges once members of the social sciences elite—Fellows in the social sciences division of the British Academy—are considered. In the class origins and education of its members, this elite becomes steadily less similar to the humanities elite and more similar to the natural sciences elite. And of particular interest is that this change is most marked in the two sections of the social sciences division of the Academy that can be regarded as closest to the notional border separating the social and the natural sciences: that is, the sections of Anthropology and Geography and of Psychology, Sociology, Demography and Statistics.

The extent of the structured social heterogeneity that now exists within the Academy could be summed up as follows. In the cohort born from 1950 onwards, Fellows in the above‐mentioned two sections have less than a 1 in 3 probability of coming from Class 1 origins and of being privately schooled and only a 2 in 5 probability of having attended Cambridge or Oxford. In contrast, Fellows in the humanities sections that are perhaps most representative of Snow's ‘traditional culture’—Classics and Archaeology, Cultural History and Philosophy—have around a 1 in 2 probability of coming from Class 1 origins and of being privately schooled and a 3 in 5 probability of being Cambridge or Oxford alumni or alumnae.

The foregoing results are reflected in the routes followed into elite positions, so that it is again the humanities elite that stands apart. With little change over time, the members of this elite are more likely than members of the natural sciences and social sciences elites to have come from Class 1 origins and to have then followed routes from private schools to university careers at Cambridge or Oxford. Conversely, members of the natural sciences and social sciences elites are more likely to have come from less advantaged class origins and to have then moved on from state schools to non‐Oxbridge university careers. And, within the Academy, clear differences emerge in the routes followed into the Fellowship as between those in the humanities division and those in the Anthropology and Geography and the Psychology, Sociology, Demography and Statistics sections of the social sciences division.

The second aim of the paper was to place our findings in the context of the atypical situation that exists in the UK in that within the institutional order through which academic elite status is accorded, the social sciences are brought together with the humanities rather than with the natural sciences. Any detailed examination of how this situation came about is beyond the scope of the present paper. But what would seem clear is that it has deep historical roots. One factor of immediate importance, evident in the events of 1901‐2, was the tendency in nineteenth‐century Britain, not paralleled in most other modernising societies, for ‘science’ to be equated with natural science and for other areas of study to be regarded as essentially ‘literary’ or otherwise ‘non‐scientific’—a tendency that has in fact shown some persistence.^29^ Elsewhere, plural usages—*sciences*, *scienze, Wissenschaften*, *Vetenskaps*—were more common, together with appropriate adjectival qualifications.

Whether or not the social sciences would have fared better in Britain if, at the elite level, they had been brought within the compass of the Royal Society or of a separate social sciences academy are, like all counterfactual questions, ones to which no definitive answer is possible, and we do not pursue them.^30^ With the data at our disposal, we can also say nothing about whether elite social scientists, despite their institutional separation from elite natural scientists, are in their world‐views, values and attitudes more akin to the latter than to members of the humanities elite. But what we can say in the light of the findings we have reported is that if some ‘Snovian disjunction’ (Pynchon, 1984) at a cultural level does exist within the higher reaches of British academic life, then, as result of decisions made in 1901‐2, the line of division in its social underlay runs not so much between the Royal Society and the British Academy as through the latter body.

## Supporting information

Supporting Information S1

## Data Availability

The data underlying this article were collected by the authors. They will be placed in the public domain once the full project on British elites, of which this study forms part, is completed.

## ENDNOTES

^1^
Prosopographical studies have been widely used by historians in the study of elites (see, e.g., Stone, 1971; Keats‐Rohan, 2007; Clark, 2008) but, until recently, less often by sociologists, who have tended to rely on data sources that cannot provide information of the detailed kind for which the approach calls.

^2^
Our approach does of course make us dependent on the selection criteria and procedures of the institutions or associations in question. But in the case of both the Royal Society and the British Academy detailed information in these respects is in the public domain: http://royalsociety.org/fellows/elections and https://www.thebritishacademy.ac.uk/fellows/fellowship/elections/. In the study of academic elites specifically, another possible method of selection is by reference to citation counts. However, while this may be viable in the case of the elite within a single discipline (see e.g. Korom, 2023 on the sociological elite), it is scarcely so in a cross‐disciplinary context, chiefly because of differences in citation practice and in the frequency of multiply authored works.

^3^
Some Fellows of the Royal Society work in research institutions outside of academia, and also for firms in the private sector. Some Fellows of the British Academy work in museums and art galleries or as private scholars. But, in both cases alike, the very large majority of Fellows were at some time employed in university institutions—82% of those in the Royal Society and 94% of those in the British Academy.

^4^
For example, the US National Academy of Sciences (founded 1863), the German Academy of Sciences (formerly the Leopoldina Academy) founded 1652 and the Royal Swedish Academy of Sciences (founded 1739) cover both the natural and the social sciences. The Italian Accademia Nazionale Reale dei Lincei, (founded 1603) and the academies established in countries in the Soviet bloc after World War II cover the whole disciplinary range.

^5^
The closest approximation is perhaps the study of Brint et al. (2020) of widely defined US cultural elites, which includes discussion of the university backgrounds of ‘prestigious academics’, as defined by their memberships of the National Academy of Sciences and of other academies, but no analyses are made of their social origins or their schooling.

^6^
Snow once remarked (Ortolano, 2009, p. 33) that in the course of his interviewing he had come to know as many scientists as anyone in the world.

^7^
Snow himself was the son of a factory clerk, attended Leicester Grammar School and started his university career at University College, Leicester.

^8^
This would appear to have been a study carried out by the Nobel Laureate in Physics, Sir Lawrence Bragg, who was a strong supporter of Snow (see Cole, 2016).

^9^
Several commentators have overlooked Snow's discussion of the social underlay of the two cultures or have mistaken Snow's views in this regard. For example, Lepenies (1988, pp. 155–7) claims that Snow saw ‘the two intellectual camps’ as being ‘almost indistinguishable’ in social class, and while noting Snow's observations on the possibility of a ‘third culture’ arising among ‘the sociological intelligentsia’, Lepenies does not mention that Snow envisaged the bearers of this culture as being socially more similar to natural scientists than to leading literary figures.

^10^
This account relies primarily on The British Academy (1903‐4). Collini (2006, p. 178) comments that the establishment of the Academy marked ‘symbolically as much as actually, a significant step towards the assertion by an intellectual or scholarly stratum of a certain kind of autonomy for itself’.

^11^
The word ‘scientist’ did not of course exist at this time. According to the *Oxford English Dictionary*, it was coined by William Whewell, Master of Trinity College, Cambridge, in 1834.

^12^
The Society's original title (still retained for official purposes) was The Royal Society of London for Improving Natural Knowledge. But ‘Natural’ here would appear to refer to the kind rather than to the object of knowledge: that is, to knowledge obtained by the natural methods of observation, experiment and calculation rather than in some way deriving from pure speculation or authority.

^13^
It does seem that this ‘marriage’ came about for purely negative reasons—because neither economics nor law could be readily fitted into the range of historical, philosophical or philological studies. In continental European academies, law would appear to have been usually allied with political science which in Britain at the time in question, scarcely existed, at least under that name.

^14^
Over the same period, the gender difference in those elected, which had previously been very wide, with men accounting for more than 90% of all elections up to the 1990s, also narrowed, and by the second half of the 2010s had almost disappeared (see Appendix 2). Age at election has been increasing from an average of 44 in the 1940s to currently 58, which is the same as for the Royal Society.

^15^
Cases are known where individuals working in these fields have declined the offer of an Academy Fellowship for fear this might prejudice their chances of being elected to the Royal Society. The number of those who are both FRS and FBA is very small, one notable instance being that of David Glass (1911–78), a distinguished demographer and Professor of Sociology at LSE.

^16^
For Fellows in more recent cohorts, *Who's Who* becomes a less reliable source of information, chiefly because of the increasing number of individuals—around 20% of those born after 1950—who apparently decline entry.

^17^
If we were to suppose that our coverage amounts to an 78% random sample of our population (which it is not) and sought to engage in inference from sample to population, we would need to include some major finite population corrections, since 78% is such a large proportion of the whole (Cochran, 1977). We cannot see that, in the present context, to proceed in this way would be at all illuminating.

^18^
Where we have information that allows us to establish the class positions of an individual's father and mother and these differ, we apply the ‘dominance’ method (Erikson, 1984). In fact, given the historical period covered, many mothers were not in employment and those who were typically held class positions that were at a lower hierarchical level within NS‐SEC than those of their husbands. Thus, in effect, parental class does in the very large majority of cases refer to father's class.

^19^
It might be asked why we do not also give here similar distributions by section for Fellows of the Royal Society. These are in fact available in previous publications (Bukodi et al., 2022, 2023) and show little variation over time. However, *chemists* are persistently more likely than other Fellows to be of relatively disadvantaged class origins—and to have been state rather than privately educated.

^20^
The gender differences shown up are, on average, relatively small (see Appendix 7.1). They indicate a tendency for women across all three elites to have a higher probability than men of coming from higher professional families rather than being of any other class origins. However, this probability declines across cohorts, and in the social sciences elite in particular (see Appendix 7.2).

^21^
The Clarendon Schools are the nine ‘public schools’ whose finances and management were investigated by the Clarendon Commission (1861–4) on account of what was taken to be their national importance as the ‘chief nurseries’ for ‘every profession and career’ in the country. They are Charterhouse, Eton, Harrow, Merchant Taylors', Rugby, St Paul's, Shrewsbury, Westminster and Winchester. Within all three elites, around a half of all those who went to Clarendon schools were at St Paul's, Westminster or Winchester.

^22^
The main gender effect shown up under the model (see Appendix 9) is that women are less likely than men to have been privately rather than state educated, with the major difference coming in attendance at the Clarendon schools, which over most of the period covered were not co‐educational—and some still are not.

^23^
As regards gender effects (see Appendix 11), the main differences are that women have a clearly lower probability than men of attending Cambridge—but not Oxford—and a higher probability of attending London.

^24^
The fact that almost a third of those in the Cultural History section of the Academy did postgraduate work at London University reflects the presence there of distinguished specialist institutions in this field, such as the Courtauld Institute of Art and the School of Oriental and African Studies.

^25^
The gender differences that are shown up under the model are the same, though somewhat weaker, as with undergraduate universities.

^26^
In the case of the Other UK undergraduate‐postgraduate sequence, the proportion of elite members staying at the same university, rather than moving on to another in this same category, is smaller than that shown for Cambridge and Oxford in Table 7 but is still 65% in the natural sciences and humanities elites, while falling to 50% in the social sciences elite. As shown by In and Breen (2023), there is in general in the UK a very strong ‘path dependency’ between individuals' universities of undergraduate and postgraduate study.

^27^
It may further be noted from Figure 6 that members of the humanities elite of all class origins are more likely than members of the two other elites to have moved on from private schooling to the Cambridge and Oxford university sequences. This may reflect the fact of superior teaching in the humanities in private schools, whereas in the case of the natural sciences teaching in state grammar and technical schools was widely regarded as being superior at least up to the 1960s (see further Bukodi et al., 2022; Turner, 2015).

^28^
As far as gender differences are concerned, there is a tendency for women across all three elites to have a higher probability than men of following non‐Oxbridge routes, regardless of the type of secondary school they attended (see Appendix 15).

^29^
For example, in 1981 a review initiated by the Conservative Education Secretary, Sir Keith Joseph, led to the UK Social Science Research Council, established in 1965 by a Labour government, being renamed, with ‘science’ symbolically dropped, as the Economic and Social Research Council.

^30^
As indicative perhaps of some dissatisfaction with existing arrangements, a separate Academy of Social Sciences was in fact formed in 2007 on the basis of the former Association of Learned Societies in the Social Sciences, dating from 1982. However, this Academy can scarcely compete with the British Academy in terms of prestige, political influence and, above all, financial resources.
