# Supplementary material for: Variation in the social composition of the UK academic elite: The underlay of the two—or three—cultures?
Source: Br J Sociol. 2024 Oct 22;76(2):201–25. doi: 10.1111/1468-4446.13154 (PMC11890434; doi:10.1111/1468-4446.13154)
Supplement: Supplementary file 1 — Supporting Information S1 [file BJOS-76-201-s001.docx]

Variation in the social composition of the UK academic elite: The underlay of the two – or three – cultures?

Appendices
